# The Fantastic Laboratory of Dr. Weigl: How Two Brave Scientists Battled Typhus and Sabotaged the Nazis

**DOI:** 10.3201/eid2111.150926

**Published:** 2015-11

**Authors:** Chong-Gee Teo

**Affiliations:** Centers for Disease Control and Prevention, Atlanta, Georgia, USA

**Keywords:** history, typhus, vaccine, Rickettsia, Weigl

Allen’s narrative is set in eastern Europe just before and during the Second World War. The subject is typhus and 2 microbiologists who were engaged frenetically in producing vaccines against it. 

The lives of the microbiologists—Rudolf Weigl, a German zoologist, and Ludwig Fleck, a Jewish physician—intersected in Lwow, eastern Poland. When the First World War broke, Weigl, stationed nearby to lead a laboratory to control typhus among Russian prisoners of war, recruited Fleck, then a student.

*Rickettsia prowazekii* had just been identified as the agent of typhus. Its propagation required that lice feed on infected humans. Reckoning that the rectal lining of the louse is chitinous and so can withstand mechanical trauma, Weigl inoculated lice anally with these rickettsiae. The experiment worked. When he inadvertently jabbed his hand with contaminated glassware and fever later arose, he had his wife place lice on him to feed. After he recovered, Weigl inoculated, successfully, rickettsiae from the lice that had fed on him to other lice. Breakthrough: propagating *R. prowazekii *without using humans was now possible.

After the Bolshevik revolution, Weigl returned to Lwow to produce vaccine by attenuating *R. prowazekii* through passage in lice and animals. To scale up vaccine production, Weigl resumed using humans to feed lice. With as many as 40 cages strapped to the leg, a person can feed 25,000 lice a month.

Fleck, too, returned to Lwow. When German troops took over this area, thousands of Jews were dispossessed and killed. Fleck’s discovery of rickettsial antigens in urine—which could be put to diagnostic use—saved his life. Retained to direct the sanitation laboratory, he contemplated using the antigens as vaccine, but that proved to be a nonstarter: to inoculate Aryans with an excretal product of diseased non-Aryans was unconscionable to his Nazi controllers.

The typhus menace continued to beset German-occupied Europe. Weigl, imposed upon to scale up his vaccine for the troops, expanded the number of louse feeders. Being a feeder became a much sought-after occupation, and louse feeding became a cover for underground operations.

Fleck was transferred to Buchenwald, where the Gestapo was meting out the now infamous atrocities to its inmates, and where vaccine production from *R. prowazeckii* harvested from rabbit lungs was to begin. His laboratory staff were an incompetent lot, unable to isolate, let alone identify, rickettsiae. Nonetheless, Fleck considered that production of a fake vaccine might be an effective way to undermine the Nazi war effort. The scam operation went ahead, and useless vials of rabbit-lung extracts were delivered to the front. The ruse was never discovered.

After liberation, the Soviets occupied Poland. Weigl, duly taking up an academic position, soon faced a contretemps: an apparatchik of mediocre accomplishments denounced him as a Nazi collaborator. Fleck’s ending was more salubrious. Assuming a microbiology professorship, then testifying in the Nuremberg trials, he finally emigrated to Israel.

Allen’s narrative is well documented, written rivetingly for general readers. They will assimilate, become reminded of, and appreciate how acts of human depravity can generate devastations of catastrophic proportions. Of comfort is that other human actions —the noble ones—can go some way to contain, mitigate, and repair the damages inflicted.

